# Detection of Antibodies to *Ehrlichia* spp. in Dromedary Camels and Co-Grazing Sheep in Northern Kenya Using an *Ehrlichia ruminantium* Polyclonal Competitive ELISA

**DOI:** 10.3390/microorganisms10050916

**Published:** 2022-04-27

**Authors:** Marisol Collins, Collins Ngetich, Milton Owido, Dennis Getange, Robert Harris, Joel L. Bargul, Boku Bodha, Daniel Njoroge, Dishon Muloi, Dino J. Martins, Jandouwe Villinger, Naftaly Githaka, Matthew Baylis, Eric M. Fèvre, Esther Kanduma, Mario Younan, Lesley Bell-Sakyi

**Affiliations:** 1 Institute of Infection, Veterinary and Ecological Sciences, University of Liverpool, Liverpool L3 5RF, UK; Marisol.Collins@liverpool.ac.uk (M.C.); hlrhar10@liverpool.ac.uk (R.H.); D.Muloi@liverpool.ac.uk (D.M.); baylism@liverpool.ac.uk (M.B.); Eric.Fevre@liverpool.ac.uk (E.M.F.); 2 International Livestock Research Institute, Nairobi P.O. Box 30709-00100, Kenya; c.ngetich@cgiar.org (C.N.); g.owido@cgiar.org (M.O.); N.Githaka@cgiar.org (N.G.); 3 Department of Biochemistry, Jomo Kenyatta University of Agriculture and Technology, Nairobi P.O. Box 62000-00200, Kenya; gdennoh89@gmail.com (D.G.); jbargul@jkuat.ac.ke (J.L.B.); 4 International Centre of Insect Physiology and Ecology, Nairobi P.O. Box 30772-00100, Kenya; jandouwe@icipe.org; 5 Directorate of Veterinary Services, County Government of Marsabit, Marsabit P.O. Box 384-60500, Kenya; bokubodha@gmail.com; 6 Department of Biochemistry and Molecular Biology, Faculty of Science, Egerton University, Njoro P.O. Box 536-20115, Kenya; edknjoroge@yahoo.com; 7 Mpala Research Centre, Nanyuki P.O. Box 555-10400, Kenya; director@mpala.org; 8 Department of Biochemistry, Faculty of Science and Technology, University of Nairobi, Nairobi P.O. Box 30197-00100, Kenya; ekanduma@uonbi.ac.ke; 9 Food and Agriculture Organisation of the United Nations (FAO), Programme and Operational Support to Syria Crisis, UN Cross-Border Hub, Gaziantep 27010, Turkey; younanmario5@gmail.com

**Keywords:** *Ehrlichia*, heartwater, camel, sheep, Kenya, *Candidatus* Ehrlichia regneryi, serosurvey

## Abstract

A disease with clinical and post-mortem presentation similar to those seen in heartwater, a tick-borne disease of domestic and wild ruminants caused by the intracellular bacterium *Ehrlichia ruminantium*, was first reported in dromedary camels in Kenya in 2016; investigations carried out at the time to determine the cause were inconclusive. In the present study, we screened sera from Kenyan camels collected before (2015) and after (2020) the 2016 disease outbreak for antibodies to *Ehrlichia* spp. using an *E. ruminantium* polyclonal competitive ELISA (PC-ELISA). Median antibody levels were significantly higher (*p* < 0.0001) amongst camels originating from areas where the heartwater-like disease was reported than from disease-free areas, for animals sampled in both 2015 and 2020. Overall median seropositivity was higher in camels sampled in 2015 than in 2020, which could have been due to higher mean age in the former group. Camels that were PCR-positive for *Candidatus* Ehrlichia regneryi had significantly lower (*p* = 0.03) median antibody levels than PCR-negative camels. Our results indicate that Kenyan camels are frequently exposed to *E. ruminantium* from an early age, *E. ruminantium* was unlikely to have been the sole cause of the outbreak of heartwater-like disease; and *Ca*. E. regneryi does not appreciably cross-react with *E. ruminantium* in the PC-ELISA.

## 1. Introduction

Dromedary camels (*Camelus dromedarius*) are important food-producing animals in northern Kenya. Their population has been steadily increasing year-on-year [1] and stood at nearly 4.7 million in 2020 [2]. The Kenyan camel meat and milk industry was estimated in 2015 to be worth approximately USD 11 million annually [3], and camels are vital for food security in the vulnerable pastoralist economy. Outbreaks of a severe disease in camels, with clinical signs similar to those seen in domestic ruminants suffering from heartwater, were recorded in northern Kenya (Garissa, Wajir, Mandera and Marsabit Counties) in 2016 [4]. The disease was characterised by sudden onset, lethargy, excitability, head pressing, aimless wandering, recumbency, rapid breathing, extreme respiratory distress, and mortality close to 100% in adult animals in the absence of antibiotic treatment. Post -mortem examination revealed pulmonary oedema, pleural exudate, hydrothorax, hydropericardium, ascites, an enlarged “cooked” liver, and nephrosis and blood in the abomasum and intestine [4]. The disease appeared to be novel; experienced camel pastoralists did not have any ‘technical term’ in the vernacular for the clinical picture presented [4]. Investigations carried out in Wajir and Marsabit counties [4,5] pointed to the possible involvement of a tick-borne pathogen (TBP) in this disease syndrome. Clinical and post-mortem signs indicated that heartwater, a fatal disease of domestic and wild ruminants transmitted by *Amblyomma* spp. ticks [6], but almost unknown in camels, was a possible candidate for the causative agent of this disease, or at least could play a role in the syndrome. Nervous signs and presence of hydropericardium and hydrothorax are characteristic of heartwater in domestic ruminants [6,7], and one or more of these manifestations were recorded in most of the affected camels, as well as heavy tick infestations [4]. Heartwater, affecting cattle, sheep, and goats, was first reported to be widely distributed in the Kenyan Highlands nearly a century ago [8]. 

The causative agent of heartwater is the obligate intracellular rickettsial pathogen *Ehrlichia ruminantium* [9], previously known as *Cowdria ruminantium* [10] and originally as *Rickettsia ruminantium* [11]. In the mammalian host, *E. ruminantium* invades neutrophils and vascular endothelial cells where it forms characteristic membrane-bound colonies or morulae in the cytoplasm [6,11]. *E. ruminantium* was isolated from *Amblyomma* spp. ticks collected at sites across Kenya [12]. Unfortunately, neither of the previous investigations of heartwater-like disease in Kenyan camels was able to confirm involvement of *E. ruminantium* by gold-standard classical microscopy (presence of *Ehrlichia* morulae in capillary endothelial cells in Giemsa-stained brain crush smears or impression smears from other internal organs) or PCR amplification of *E. ruminantium* DNA in camel samples. However, sequencing of Anaplasmataceae 16S rRNA PCR products amplified from blood collected from diseased camels in 2016 [4] indicated the presence of DNA of two *Ehrlichia* spp. closely related to *E. ruminantium*: *Candidatus* Ehrlichia regneryi [13] and *Ehrlichia canis*. Possible heartwater has only been described convincingly in a single camel in Sudan over 60 years ago [14]; more recently, an outbreak affecting nearly 10% of 500 camels was reported from Chad [15], but diagnosis of heartwater was based on clinical signs and microscopy and no details were presented. 

We recently carried out a study investigating the incidence of TBP in camels and ticks feeding on them in areas of Marsabit County, northern Kenya [16]. Using molecular assays, we detected *Ca*. E. regneryi in jugular blood of 43/296 healthy camels (14.5%), and *E. ruminantium*, *Ca*. E. regneryi, *Ehrlichia chaffeensis*, and an unidentified *Ehrlichia* sp. in ticks removed from the camels [16]. 

In the present study, serum samples collected from the same camels were tested for antibodies to *E. ruminantium* and other *Ehrlichia* spp. using a polyclonal competitive ELISA (PC-ELISA) that can be applied to multiple host species simultaneously without the need for species-specific reagents [17]. Camels in areas where the heartwater-like disease had and had not been reported were sampled for comparison and, where available, co-grazing sheep were also sampled. Sheep are good sentinels for the presence of *E. ruminantium* as, following natural exposure to the pathogen, they retain very high antibody levels for prolonged periods [18]. To obtain a statistically stronger picture of seropositivity related to location, age, and gender, we also tested sera collected from camels maintained in experimental herds belonging to two research institutes located in Laikipia and Machakos counties in areas of mixed-use land systems dominated by livestock production, where heartwater-like disease in camels has never been reported. To further investigate the possible exposure of camels to *E. ruminantium* prior to the 2016 outbreaks of heartwater-like disease [4], we tested a panel of archived sera collected in 2015 from peri-urban camel herds in Isiolo County where heartwater-like disease in camels has never been reported, and from camels brought from different areas of northern Kenya for slaughter at a site in Machakos County. Finally, we investigated whether any correlation could be found between PC-ELISA antibody levels and the presence or absence of *Ca*. E. regneryi DNA reported previously [16] in the blood of the camels sampled in Marsabit County.

## 2. Materials and Methods

### 2.1. Sample Collection

**Figure 1:**
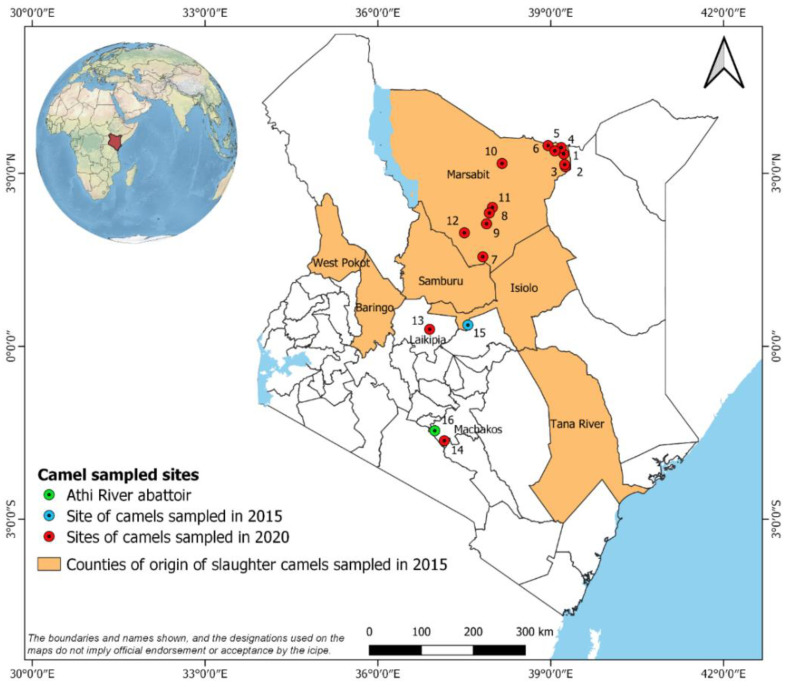
Map of Kenya showing sampling sites in Marsabit, Laikipia, and Machakos Counties visited in 2020 (red spots), sampling site in Isiolo County visited in 2015 (blue spot) and site of the Athi River abattoir (green spot). Counties of origin of camels sampled in 2015 are shaded in brown. Locations of numbered sites are listed in Tables S1 and S2.

The study area in Marsabit County, northern Kenya, has been described previously [16]. Six field sampling sites visited in February 2020 were located in areas where heartwater-like disease had been reported in camels (Bori, Yaballo, Misa, Dabel, Funanyatta and Gola), while the remaining six field sites were located in areas unaffected by the disease (Laisamis, Korr, Kamboe, Hula Hula, Burgabo and Shegel) (Table S1, Figure 1). Two additional sites visited in October 2020 in areas unaffected by heartwater-like disease in camels were located at Mpala Research Centre, Laikipia County, and the International Livestock Research Institute (ILRI) Kapiti Research Station, Machakos County (Table S1, Figure 1). Between seven and 16 dromedary camels aged between 2 months and 30 years (median age 9 years) were sampled from available herds at each site (total 306 camels); where available, co-grazing adult sheep (total 90 sheep) were also sampled as indicators for the presence of *E. ruminantium* infection in an area because they develop high and long-lasting levels of serum antibodies following exposure [18,19]. Age and gender of sampled animals was recorded (Table S1). Jugular blood (4 mL) was collected by venepuncture into serum vacutainer tubes containing a coagulation activator (BD Diagnostics, Oxford, UK). Blood samples were stored at 4–10 °C for a maximum of 6 h; serum was manually separated and stored in liquid nitrogen for transport to Nairobi, where they were stored at −80 °C until further analysis. 

The archived camel sera (total 373 camels) were collected in 2015 and transported as described above and stored at −80 °C. They comprised samples collected from randomly-selected animals in 16 different herds maintained in the peri-urban surroundings of the town of Isiolo, Isiolo County, and from all camels brought for slaughter at the Athi River slaughterhouse, Machakos County, over a 10-day period (Table S2, Figure 1). Age and gender of all camels, and the geographic origins of the Athi River camels, were recorded (Table S2).

### 2.2. Ethical Approval

The animal sampling in Marsabit and at Mpala Research Centre was carried out following the experimental guidelines and procedures approved by the University of Nairobi Biosafety, Animal Use and Ethics Committee (Ref: FVM BAUEC/2019/200) and Kenya’s National Commission for Science, Technology and Innovation (NACOSTI) (Ref: NACOSTI/P/19/72855/27325). Care was taken to minimise discomfort when collecting blood samples from camels and sheep. Members of the sampling team informed the camel pastoralists of the reasons for the study in their own language, and animal sampling was conducted after receiving verbal consent, as most herders were unable to read or write. The sampling at Mpala Research Centre was approved based on best-practice protocols and was carried out under supervision of the Mpala–Kenya Wildlife Service veterinarian; The Mpala Research Centre is registered with NACOSTI. For the Kapiti Research Station, Isiolo and Athi River sampling, camel samples were obtained under the approval of the ILRI Institutional Animal Care and Use Committee (reference ILRI-IACUC2015.01/01) and permits were obtained from the Directorate of Veterinary Services. The ILRI Institutional Research Ethics Committee is registered and accredited by NACOSTI. 

### 2.3. E. ruminantium PC-ELISA

The PC-ELISA was carried out as described previously [17,20] with minor modifications. Soluble antigens of *E. ruminantium* (Welgevonden strain) elementary bodies (EB) were isolated from infected bovine pulmonary artery endothelial cells. Immulon™ IB 96-well plates (Thermo Fisher, Loughborough, UK) were coated with 100 µL/well of EB antigen diluted 1:1000 in carbonate-bicarbonate buffer (Sigma Aldrich, Gillingham, UK), leaving duplicate uncoated wells as a blank control, and incubated overnight at 4 °C. Manual plate washing with 300 µL/well phosphate-buffered saline with 0.05% Tween (PBST) was repeated five times between steps. Aliquots of 50 µL of undiluted test or control sera were added to duplicate wells, with 50 µL of PBS added to duplicate competitor control and no-antigen control wells. Within 5 min of serum addition, 50 µL of biotinylated competitor antibody diluted 1:400 in PBS with 0.1% Tween was added to all wells and plates incubated for 1 h at 37 °C, then washed as before. ExtrAvidin-peroxidase conjugate (Sigma Aldrich, Gillingham, UK) diluted 1:2500 with PBST was added to each well and plates incubated for 30 min at 37 °C, then washed as before. Tetramethyl benzidine substrate (Sigma Aldrich, Gillingham, UK) was dissolved in phosphate-citrate buffer (Sigma Aldrich, Gillingham, UK), 100 µL was added to all wells, and plates were incubated for 20 min in the dark at room temperature. The reaction was terminated by adding 50 µL of 2 M sulphuric acid to all wells. The plates were read at 450 nm on an Infinite^®^ F50 Robotic Plate Reader (Tecan, Männedorf, Switzerland) or an iMark™ Microplate Absorbance Reader (Bio-Rad, Watford, UK). A panel of nine presumed *E. ruminantium*-negative camel sera collected from healthy animals in Libya [21] where *Amblyomma* spp. ticks or heartwater have never been reported [6,22], and stored thereafter at −80 °C, were used to determine the range of percentage inhibition (PI) values expected for healthy, naïve camels. Serum collected from a sheep recovered from experimental infection with *E. ruminantium* (Sankat 430 strain) and challenged with *E. ruminantium* (Gardel strain) [23] was used as a positive control. A panel of 12 sera collected from naïve UK sheep in 2019 were used as negative controls. Inhibition levels of test and control sera, expressed as percentage inhibition (PI) (following automatic deduction of the no-antigen control OD value) were calculated as follows:

PI = 100 − ([100 × mean test serum OD value]/mean competitor control PBS OD value)

### 2.4. Statistical Analysis

Our sample size calculation for camels was based on limited available knowledge of heartwater-like disease outbreaks to date, including an unknown degree of clustering at the herd level. During studies of outbreaks, up to 40% of camels in a herd were affected [4] and we therefore set the intra-class correlation coefficient (ICC) to 0.5. For logistical reasons, it was deemed feasible to sample eight camels per herd in affected areas. Using the equation design effect (DEFF) = 1 + ICC(N − 1), where N = sample size per cluster, this gives a DEFF of 4.5. Mortality from heartwater-like disease in affected herds, to which treatment with tetracycline was applied early, was reported as 7.5% [4]. We therefore took this as the estimate of prevalence, assuming that camel herders may have been trying to protect their animals with tetracycline (if available). Using a DEFF of 4.5 and estimated prevalence of heartwater-like disease of 0.075, we calculated sampling 120 camels (15 herds, 8 camels per herd) to be 95% confident that our sample prevalence fell within 10% of the true prevalence. For comparison of affected and unaffected sites, a further 120 camels (15 herds, 8 camels per herd) were sampled. Where present, up to eight co-grazing sheep per camel herd were sampled. 

Field and laboratory data were collated and stored in Microsoft Excel. Statistical analysis was performed in Stata 14 (Statacorp LLC, College Station, TX, USA) and GraphPad Prism v.9 (GraphPad Software, La Jolla, CA, USA). Descriptive data are presented as count, percentage, mean (95% CI) or median (interquartile range [IQR]). Continuous data sets were assessed for normality using the Shapiro–Wilk test and inspection of Q–Q plots. Effect of independent variables (sampling site, area with/without reported heartwater-like disease, sex, age, and molecular detection of TBP in blood) on the dependent variable (PI) was analysed using Kruskal–Wallis test, Mann–Whitney U test, and Spearman’s correlation coefficient (*rho*). For all analyses, values of *p* ≤ 0.05 were considered significant.

## 3. Results

### 3.1. Application of the PC-ELISA for Detection of Exposure of Camels to Ehrlichia spp. 

The *E. ruminantium* PC-ELISA PI values for control naïve Libyan camels ranged between 3 and 30, and the values for naïve UK sheep fell between 14 and 39. These values fell well within the range of values for negative ruminant sera recommended for use with the PC-ELISA [17], confirming the likely absence of non-specific cross-reactivity in the test with antibodies in camel sera to non-ehrlichial agents. 

**Figure 2:**
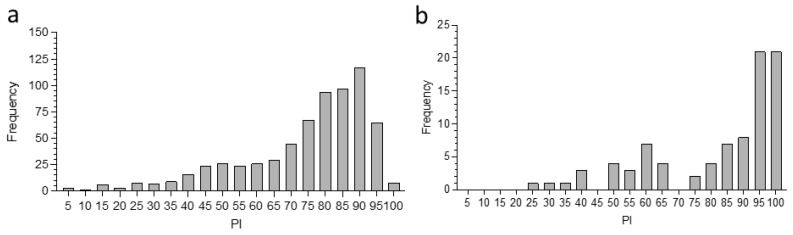
Distribution of PC-ELISA percentage inhibition (PI) values for serum from all camels and sheep sampled during the present study at sites in Marsabit, Laikipia, and Machakos counties, and camels sampled at sites in Isiolo and prior to slaughter at Athi River in 2015; (**a**) camels (*n* = 679); (**b**) sheep (*n* = 90).

In total, 396 serum samples were collected and tested by PC-ELISA from 286 camels and 74 co-grazing sheep in 37 herds at the 12 sites in Marsabit County, 8 camels and 8 co-grazing sheep at Mpala Research Centre, and 12 camels and 8 co-grazing sheep at ILRI Kapiti Research Station (Table S1). A further 143 and 230 archived sera from peri-urban camels sampled at Isiolo and camels sampled prior to slaughter at Athi River, respectively, were tested (Table S2). Figure 1 shows the overall distribution of PI values for camels (Figure 2a) and co-grazing sheep (Figure 2b) sampled at all sites in Kenya. While the camel sera did not show any clear division between likely negative and likely positive groups, the majority of PI values fell in the range 70–100, indicating previous exposure to at least one *Ehrlichia* sp. In contrast, the sheep sera fell into two categories, with PI < 65 and PI > 75; in the latter category, the majority of samples showed PI values in the range >90, indicating previous exposure to *E. ruminantium* [17]. 

### 3.2. PC-ELISA Seropositivity in Camels Related to Age and Sex

**Figure 3:**
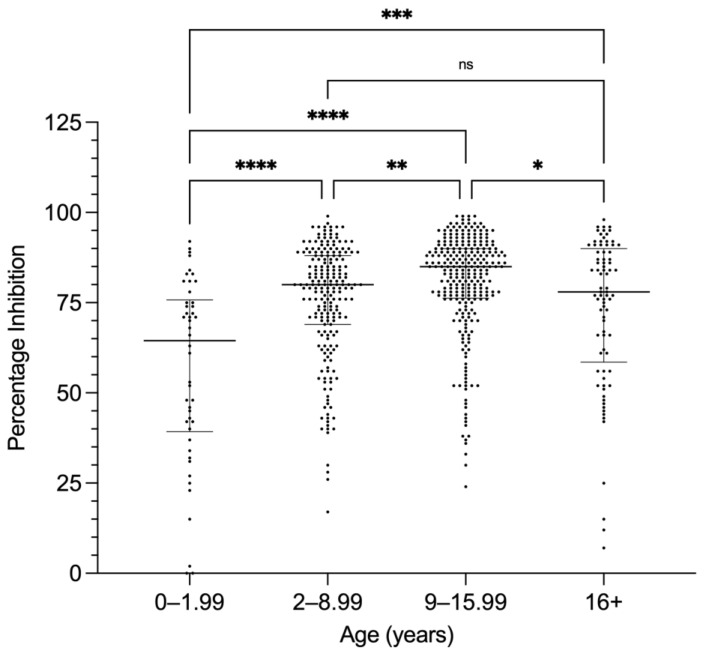
Distribution of PC-ELISA percentage inhibition (PI) values according to age groups (0–1.99 years, 2–8.99 years, 9–15.99 years, and 16+ years) of all camels sampled in Marsabit, Laikipia, and Machakos counties in 2020 (*n* = 284) and at Isiolo and prior to slaughter at Athi River in 2015 (*n* = 323) for which age was recorded. Median PI (widest horizontal line) increased significantly with age up to 15.99 years, thereafter showing a small but significant decrease. Data were analysed by a Kruskal–Wallis test, with Dunn’s post hoc test for pairwise comparisons. * *p* = 0.0294, ** *p* = 0.0011, *** *p* = 0.0003, **** *p* < 0.0001.

Seropositivity in the PC-ELISA has previously been reported to increase with age [18,24]. The ages of camels sampled during the present study and in 2015, and whose ages were recorded (*n* = 607), ranged from a minimum of 2 months (0.17 years) to a maximum of 30 years. The camel samples were grouped into four age ranges (0–1.99, 2–8.99, 9–15.99, and 16+ years) and the distribution of PI values obtained in the PC-ELISA is shown in Figure 3. The increase in median PI between the first and second groups was highly significant (*p* < 0.0001), and moderately significant (*p* = 0.0011) between the second and third groups. Thereafter, median PI showed a small but significant decline in camels aged between 16 and 30 years (Figure 3). Results of a Spearman’s correlation test indicate a significant relationship between age and PI (rs = 0.16, *p* < 0.0001).

There are no reports of differences between seropositivity of male and female animals for either the PC-ELISA or other assays for antibodies against *E. ruminantium*. Amongst the 636 camels for which sera were tested using the *E. ruminantium* PC-ELISA and for which sex was recorded, the median PI of the 161 male camels was 79% (IQR 27, range 0–107%) and the median PI of the 475 female camels was 81% (IQR 22, range 2–115%) (Figure S1). In a two-tailed Mann–Whitney test, the difference in PI between male and female camels was not significant (*p* = 0.16).

### 3.3. Comparison between Animals Originating from Areas with and without Reported Heartwater-like Disease in Camels

**Figure 4:**
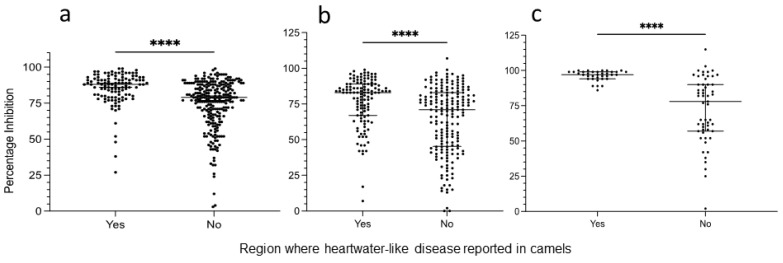
Comparison of PC-ELISA percentage inhibition (PI) values for camels and sheep collected in or originating from areas of Kenya where heartwater-like disease in camels was reported in 2016 (Yes) with areas of Kenya where such disease was not reported (No); (**a**) camels sampled in 2015 at Isiolo (*n* = 143) and Athi River slaughterhouse (*n* = 230); (**b**) camels sampled during the present study in 2020 in Marsabit, Laikipia, and Machakos counties (*n* = 306); (**c**) sheep sampled during the present study in 2020 (*n* = 90). In all groups, median PI was significantly higher in animals sampled in, or originating from, areas where heartwater-like disease was reported in 2016. Graphs include all data points with median value shown by widest horizontal line. Data were analysed in a two-tailed Mann–Whitney test; **** *p* < 0.0001.

PC-ELISA results for samples from camels and co-grazing sheep collected in (Table S1), or originating from (Table S2), areas where heartwater-like disease was reported in camels in 2016 and areas where such disease was not reported were compared (Figure 4). Firstly, for samples collected in 2015, prior to the first disease report, the median PI value for sera of 115 camels originating from regions where heartwater-like disease was subsequently reported was higher at 88% (IQR 12, overall range 26–99%) than that of the 258 camels originating from areas where such disease was not reported (median 79%, IQR 21, overall range 2–100%). In a two-tailed Mann–Whitney test, the difference in PI between the two groups was statistically significant (*p* < 0.0001) (Figure 4a). Secondly, for samples collected in 2020, the median PI value for sera of 122 camels originating from regions where heartwater-like disease had previously been reported was also significantly higher (*p* < 0.0001) at 83% (IQR 22, overall range 7–99%) than that of the 184 camels originating from areas where such disease was not reported (median 71%, IQR 38, overall range 0–107%) (Figure 4b). Sheep (*n* = 35) sampled in 2020 at sites reporting heartwater-like disease in camels also had a significantly higher median PI of 97 (IQR 5, overall range 86–100%) than sheep (*n* = 55) at sites not reporting disease (median PI 78, IQR 33, overall range 2–115%) (*p* < 0.0001) (Figure 4c). 

**Figure 5:**
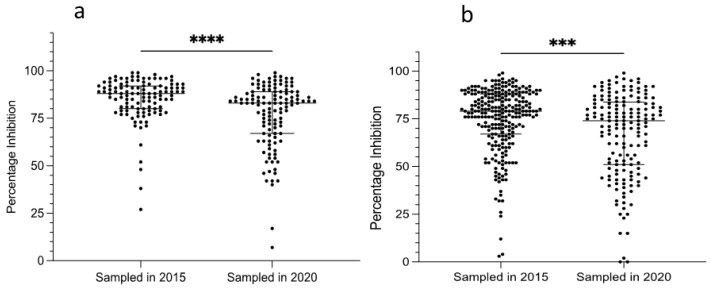
Comparison of PC-ELISA percentage inhibition (PI) values for sera of camels sampled before (2015) and after (2020) the first report of heartwater-like disease in camels in Kenya; (**a**) camels sampled in areas where the disease was reported; (**b**) camels sampled in areas where the disease was not reported. In both groups, median PI (widest horizontal line) was significantly lower in 2020 than in 2015. Data were analysed in a two-tailed Mann–Whitney test; *** *p* = 0.0002, **** *p* < 0.0001.

To investigate whether there was any difference between the levels of seropositivity before and after the first report of heartwater-like disease in camels, PI levels were compared between camels sampled in the present study in 2020 and those sampled in 2015, from areas with and without reports of the disease (Figure 5). Amongst camels sampled in, or originating from, areas where heartwater-like disease was reported in 2016 (*n* = 237), the median PI of the 115 camels sampled in 2015 (median 88, IQR 19, range 27–99%) was significantly higher (*p* < 0.0001) than that of the 122 camels sampled in 2020 (median 83, IQR 32, range 7–99%) (Figure 5a). Amongst camels sampled in, or originating from, areas where the disease was not reported (*n* = 422), the median PI of the 258 camels sampled in 2015 (median 79, IQR 21, range 3–99%) was also significantly higher (*p* = 0.0002) than that of the 164 camels sampled in 2020 (median 74, IQR 33, range 0–99%) (Figure 5b). It should be noted that the mean age of camels sampled in 2015 was higher than that of camels sampled in 2020 (Tables S1 and S2), which could have contributed to higher levels of seropositivity.

### 3.4. Relationship between PC-ELISA Seropositivity and Presence of Ehrlichia spp. DNA in Camel Blood

**Figure 6:**
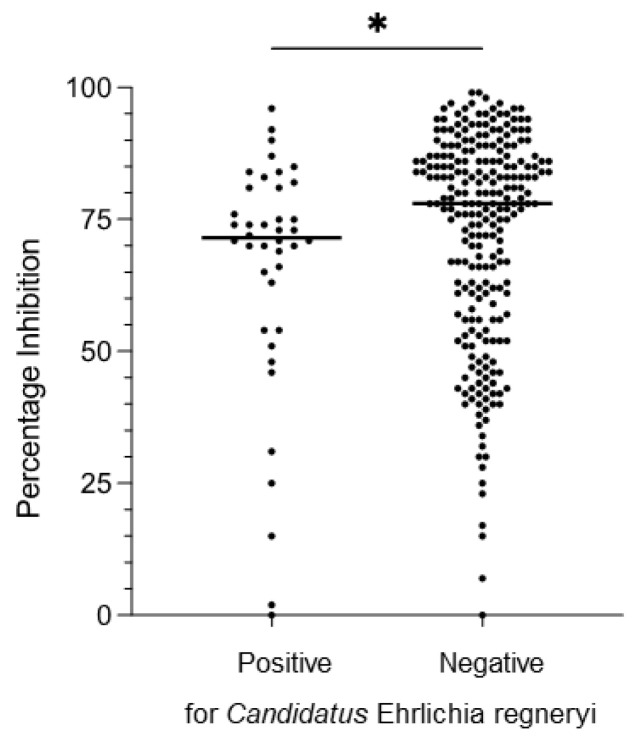
Comparison of PC-ELISA percentage inhibition (PI) values obtained from camels sampled in Marsabit County whose blood was PCR-positive (*n* = 40) or PCR-negative (*n* = 246) for *Candidatus* Ehrlichia regneryi [16]. Median PI value (widest horizontal line) for negative camels was significantly higher than that of positive camels. Data were analysed using a two-tailed Mann–Whitney test; * *p* = 0.03.

In our parallel study [16], blood of the camels sampled in Marsabit County was also screened by high-resolution melting analysis and sequencing of PCR products obtained using genus-specific primers for presence of *Ehrlichia* spp. DNA. No *E. ruminantium* DNA was detected in any of the camels, but *Ca*. E. regneryi was detected in the blood of 40 out of 286 animals for which sera were available. To investigate whether *E. ruminantium* PC-ELISA positivity was associated with presence of *Ca*. E. regneryi in blood, indicating possible cross-reactivity between the two species, we compared PI of camels that were PCR-positive or PCR-negative for *Ca*. E. regneryi (Figure 6). PI values in PCR-positive camels ranged from 0–96%, median 72% (IQR 25), while in PCR-negative camels (*n* = 246) the PI ranged from 0–99%, median 78% (IQR 28). In a two-tailed Mann–Whitney test, there was a significant difference between the two groups, with higher mean PI in camels testing negative for *Ca*. E. regneryi (*p* = 0.03) (Figure 6).

## 4. Discussion

Our study represents the first attempt to screen sera from camels for antibodies to any species of *Ehrlichia*, using an ELISA developed for detection of exposure to *E. ruminantium*. Previously, reports of presence of *Ehrlichia* spp. in camels were based on diagnosis of suspected heartwater by microscopy and clinical signs [14,15] or molecular analysis of DNA extracted from camel blood or post-mortem tissues. A small proportion (4/100) of dromedary camels sampled at an abattoir in Saudi Arabia were reported to harbour DNA of an *Ehrlichia* in spleen tissue [13]. Based on sequence analysis of fragments of the 16S rRNA and *groEL* genes, this novel *Ehrlichia* was closely related to *Ehrlichia minasensis* (first isolated in Brazil and originally named *Ehrlichia mineirensis*), a pathogen of cattle harboured by *Rhipicephalus microplus* ticks [25], and the canine pathogen *E. canis*. The novel camel *Ehrlichia*, named *Ca*. E. regneryi, was placed in a separate branch from *E. ruminantium* within the *Ehrlichia* phylogeny [13]. A subsequent study in Saudi Arabia detected a partial 16S rRNA sequence in blood of 1/170 healthy camels that was identical to *E. canis* and distinct from *Ca*. E. regneryi [26]; *E. minasensis* was not included in the phylogenetic analysis. In the first study carried out on Kenyan camels, 16S rRNA sequences with high similarity to *Ca*. E. regneryi and *E. canis* were detected in two sick animals during an investigation of heartwater-like disease in camels at two sites in northern Kenya [4]. In our recent follow-up study [16], we detected *Ca*. E. regneryi in 14.5% of healthy camels but failed to detect *E. ruminantium* or any other *Ehrlichia* spp. in camel blood. 

The indirect MAP1-B ELISA [19,27] has been widely used in serosurveys of cattle and small ruminants in several African countries and on Caribbean and Indian Ocean islands [20,28,29,30,31,32,33,34] and is considered to be highly specific for *E. ruminantium*. However, as far as we know, the specific anti-species conjugate required for the MAP1-B ELISA has not been developed for camels. The *E. ruminantium* PC-ELISA employed in the present study has the advantage that it can be applied to sera from any mammal, without the need for host species-specific reagents [17]. A comparison of the two assays using field sera from sheep and cattle in Ghana found that both were highly specific, and that the PC-ELISA gave more consistent results than the MAP1-B ELISA with bovine sera following seroconversion to *E. ruminantium* [20]. The PC-ELISA was then used in extensive longitudinal and point prevalence surveys of cattle, sheep and goats over a three-year period in Ghana [18,24] and a modified version of the assay was used in a serological survey of *E. ruminantium* exposure in small ruminants in northern Cameroon [35]. We tested a panel of nine dromedary camel sera collected from animals in Libya, where heartwater, *Ehrlichia* spp. bacteria or *Amblyomma* spp. ticks have never been reported in or on domestic animals [22,36,37], to obtain baseline data on PC-ELISA PI levels in presumed naïve camels. This gave us a potential cut-off level of 35 PI for exposure to any *Ehrlichia*, which compared well with the cut-offs of up to 50 PI for *E. ruminantium*-negative, 51–70 PI for *Ehrlichia*-positive, 71–85 PI for sera positive for *E. ruminantium* or *Ehrlichia ovis* (sheep only) and 85 PI for *E. ruminantium*-positive sera [17,18,20]. 

When applied to serum samples collected from camels during the present study and archived samples collected from camels in 2015, prior to the first report of heartwater-like disease in camels, the PC-ELISA PI values ranged from zero to >100%, but the vast majority (644/679, 94.8%) were >35, suggesting that most of the camels had been exposed to at least one *Ehrlichia* sp. Even with a more conservative cut-off point of 50 PI [17], 86.9%% (590/679) were seropositive in the PC-ELISA. Considering the most conservative, *E. ruminantium*-specific cut-off of 85 PI [17], 190 samples (28.0%) exceeded this level, suggesting that these animals were very likely to have been recently exposed to *E. ruminantium*, or an antigenically very closely-related bacterium. 

No difference was seen between PC-ELISA seropositivity levels of male and female camels; although three times as many females as males were tested, the overall sample size was sufficiently large to avoid bias. Most previous *E. ruminantium* serosurveys did not distinguish between male and female animals. Using the MAP1-B ELISA, a survey of ranch cattle in Tanzania reported higher seroprevalence amongst females than males, although the difference was not statistically significant [33]. The only previously reported gender-associated response to *E. ruminantium* infection related to pregnancy [6]; heavily pregnant *Bos taurus* cattle in South Africa were particularly prone to develop the peracute form of heartwater [7]. 

The median seropositivity level of camels increased with age up to 16 years, with a subsequent decline in older animals. A similar pattern of seropositivity rates increasing with age was reported from cattle and small ruminants sampled in Ghana [18,24], although in the former study the animals were only followed up to about 3 years of age and the latter study combined all results for animals >1 year of age. A positive correlation between age and seropositivity was also reported for cattle sampled up to 9 years of age in Tanzania [33]. In contrast, no significant association between *E. ruminantium* seropositivity and age was reported for goats (1–5 years old) or cattle (1–7+ years old) in Zimbabwe tested with the MAP1-B ELISA [31]. As seen in the present study, an increase in seroprevalence to *Coxiella burnetii* in camels up to 15 years of age, followed by a decrease in older animals, was reported from Tunisia [38]. However, significant association of seropositivity with camel age was not found for *Anaplasma* spp. in Egypt [39], or *C. burnetii* in Algeria [40], although in both studies camels over 15 years of age were not considered separately. The ability of older camels to mount a serological response to tick-associated infections and the persistence of serum antibodies in such older animals deserves further study. 

PI values were on average significantly higher for camels collected in, or originating from, areas of northern Kenya where heartwater-like disease had been reported in camels, than in areas where such disease had not been reported. This pattern was seen both in sera collected during the present study and in archived sera collected from camels in 2015, prior to the first report of the disease. However, from serology alone it was not possible to ascribe seropositivity specifically to exposure to *E. ruminantium*. Waning of antibodies to *E. ruminantium* following field exposure in cattle reported for the MAP1-B ELISA [30] and the PC-ELISA [18] could also occur in camels. The pattern of antibody production following exposure of camels to *E. ruminantium* could only be investigated by experimental infection of known naïve camels with the pathogen and following antibody levels post recovery. Interestingly, the overall levels of seropositivity were higher in camels sampled prior to the first report of heartwater-like disease in camels than in camels sampled in 2020, regardless of whether or not they originated from an area where the disease was reported. It is unclear whether this was due to the higher average age of the 2015 cohort, or to some other factor. 

The camels sampled in Marsabit County in the present study were also tested for the presence of *Ehrlichia* spp. DNA in blood [16] collected at the same time as the sera collected for the PC-ELISA analysis. While none of these camels were positive for *E. ruminantium* DNA, out of the 286 camels tested by PC-ELISA, 40 were positive for *Ca*. E. regneryi [16]. This presented an opportunity to investigate if the seropositivity detected in the PC-ELISA could be due to cross-reactivity between *E. ruminantium* and *Ca*. E. regneryi. We found a negative correlation between *Ca*. E regneryi DNA in blood and PC-ELISA seropositivity in camels; low antibody levels (<55 PI) were seen in around a quarter of the PCR-positive animals, while only 10% of these animals had PI > 85. This suggested that the high antibody levels detected in sera from the majority of PCR-negative animals resulted from exposure to another *Ehrlichia* species. However, from the data presented here and in our previous study [16], we cannot determine conclusively if this other *Ehrlichia* species was *E. ruminantium*. 

Although co-grazing sheep were only available for sampling at some of the sites, there was a much clearer distinction between seropositivity rates and PI levels between areas where heartwater-like disease had and had not been reported in camels. All sampled sheep in the former areas exhibited PI levels > 85 (*n* = 40, representing 4/5 sites), with most exceeding 95 PI. In the latter areas, less than half of the sampled sheep (20/55) exhibited PI levels > 85. However, we found *Amblyomma* spp. ticks harbouring *E. ruminantium* in both areas of Marsabit County [16]; interestingly, of the total number of *Amblyomma gemma* and *Amblyomma lepidum* ticks collected during our study, 436 were collected at the sites where heartwater-like disease had been reported in camels, while only 23 were collected from animals at sites where heartwater-like disease had not been reported in camels. Ticks in both sets contained *E. ruminantium* DNA at approximately similar rates (8.7% and 7.1%) [16]. Both species are reported to be able to transmit *E. ruminantium* to domestic ruminants [6,12]. *Amblyomma variegatum*, the species most widely-associated with heartwater in West, Central, and East Africa [6,36], was not found on camels or sheep during our survey in Marsabit County [16]. This tick species is present in other parts of Kenya [12,36,37,41,42] and was the third most common species found on camels in Isiolo [43], although heartwater-like disease was not reported from Isiolo County during the 2016 outbreak [44]. The role of different *Amblyomma* spp. in transmission of *E. ruminantium* to domestic ruminants and, potentially, camels in Kenya, remains unclear, indicating a need for further sampling and screening of both ticks and livestock.

## 5. Conclusions

The *E. ruminantium* PC-ELISA revealed high rates and levels of seropositivity in Kenyan camels, particularly amongst animals sampled in areas where heartwater-like disease had been reported in camels. Co-grazing sheep in these areas also showed extremely high rates and levels of seropositivity. However, these results alone do not provide sufficient evidence to indicate whether or not camels become infected with *E. ruminantium*. Further experimental and field studies are needed to determine whether camels are susceptible to infection with *E. ruminantium* and, if so, whether clinical disease ensues and how it is manifest. High seropositivity could indicate that, under normal circumstances, camels are exposed to *E. ruminantium* and become infected but do not suffer disease. Heartwater-like disease in camels could occur when the animals are in poor condition or subject to extrinsic factors or systemic disturbances, as discussed previously [6]), although animals involved in the 2016 outbreak were reported to be in good body condition [4]. Further studies are needed to determine the aetiology of heartwater-like disease in camels and its implications for camel health; however, our results do not suggest that infection with *Ca*. E. regneryi results either in heartwater-like disease or in high levels of serum antibodies detectable by the *E. ruminantium* PC-ELISA.

## Data Availability

All data supporting the reported results are included within this publication.

## Supplementary Materials

The following supporting information can be downloaded at: https://www.mdpi.com/article/10.3390/microorganisms10050916/s1, Figure S1: Comparison of PC-ELISA percentage inhibition (PI) values between male and female camels, Table S1: Samples collected in 2020: sampling sites in Marsabit, Laikipia, and Machakos counties, Table S2: Samples collected in 2016: site visited in Isiolo County and origins of camels sampled prior to slaughter at Athi River abattoir.
