# Supplementary material for: Detection of Antibodies to Ehrlichia spp. in Dromedary Camels and Co-Grazing Sheep in Northern Kenya Using an Ehrlichia ruminantium Polyclonal Competitive ELISA
Source: Microorganisms. 2022 Apr 27;10(5):916. doi: 10.3390/microorganisms10050916 (PMC9144424; doi:10.3390/microorganisms10050916)
Supplement: Supplementary file 1 [file microorganisms-10-00916-s001.zip › microorganisms-1688162-supplementary.pdf]

Detection of antibodies to *Ehrlichia* spp. in dromedary camels and co-grazing sheep in northern Kenya using an *Ehrlichia ruminantium* polyclonal competitive ELISA

Supplementary Figures and Tables

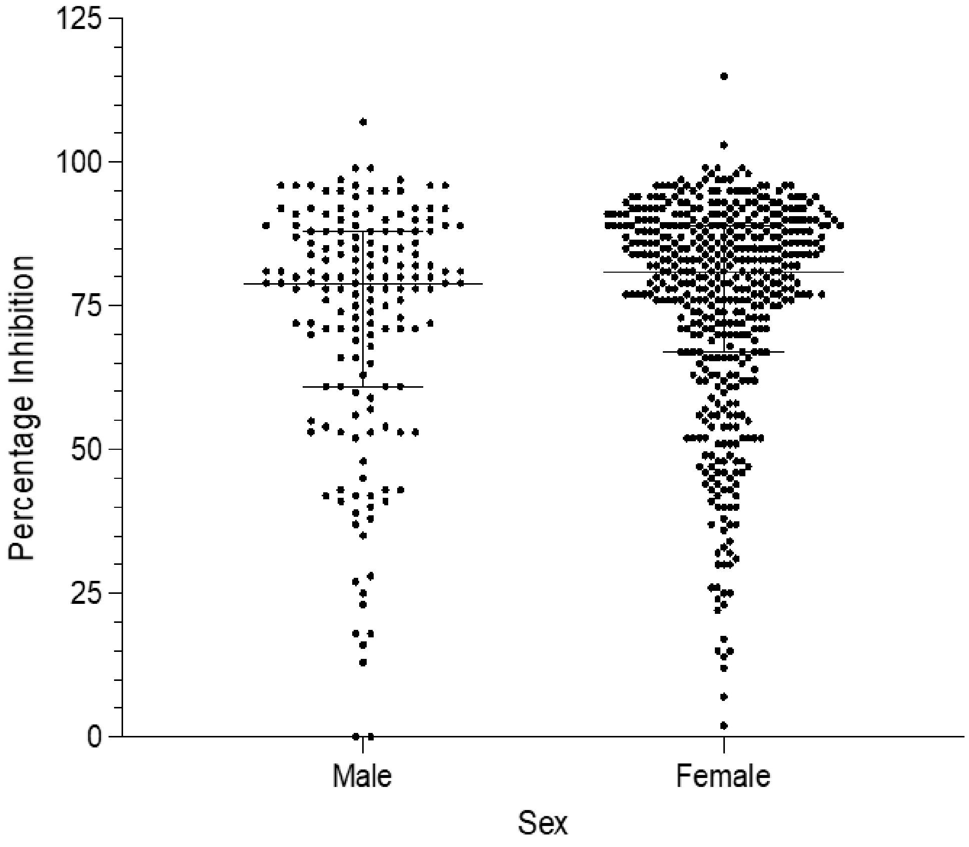

Figure S1. Comparison of PC-ELISA percentage inhibition (PI) values between male and female camels. Data was analysed for all camels for which sex was recorded, sampled during the present study in 2020 in Marsabit, Laikipia and Machakos Counties, and in 2015 in Isiolo County and prior to slaughter at Athi River, prior to the first report of heartwater-like disease in camels,. There was no significant difference in overall PI levels between male (n=161) and female (n=475) camels (two-2-tailed Mann-Whitney test, P=0.16).

Table S1. Summary of sites where camels and co-grazing sheep (if available) were sampled during the present study in 2020, showing whether or not heartwater-like disease was reported in camels at the sites in 2016, herd and animal numbers, sex and median age (interquartile range [IQR]). Sites were named after the nearest village or settlement; - = no sheep sampled; NR = not recorded

		**Camels**			**Sheep**		
**Sampling site (site no.)**	**Heartwater-like disease reported**	**Herds sampled**	**No. sampled (males/females)**	**Median age in years (IQR)**	**Flocks sampled**	**No. sampled (males/females)**	**Median age in years (IQR)**
Misa (1)	Yes	3	24 (8/16)	7.5 (6.5)	3	20 (6/14)	2.5 (2.36)
Dabel (2)	Yes	3	24 (8/16)	8 (7.75)	-	-	-
Gola (3)	Yes	3	24 (1/23)	10 (11)	-	-	-
Yaballo (4)	Yes	3	23 (5/18)	8 (11)	2	15 (4/11)	3 (1)
Funanyatta (5)	Yes	1	7 (0/7)	7 (7)	-	-	-
Bori (6)	Yes	3	20 (4/16)	8 (6.5)	-	-	-
Laisamis (7)	No	7	55 (6/49)	15 (9)	2	16 (3/13)	3 (1.25)
Hula Hula (8)	No	1	8 (1/7)	12 (12.25)	-	-	-
Kamboe (9)	No	2	16 (4/12)	4 (6.60)	1	8 (1/7)	4 (1.5)
Burgabo (10)	No	1	8 (2/6)	5 (2)	1	8 (2/6)	5 (2)
Shegel (11)	No	3	24 (11/13)	7.5 (5.25)	-	-	-
Korr (12)	No	7	53 (6/47)	12 (15)	1	7 (1/6)	3 (2)
Mpala (13)	No	1	16 (8/8)	NR	1	8 (3/5)	NR
Kapiti (14)	No	1	12 (6/6)	NR	1	8 (4/4)	NR
**Total**		37	306 (67/239)	9 (9)	11	90 (24/66)	3 (2)

Table S2. Summary of numbers of camels sampled in 2015 at peri-urban sites in Isiolo and prior to slaughter at Athi River, showing the six regions of origin for camels sampled at Athi River, whether or not heartwater-like disease was reported at the sites in 2016, animal numbers, sex and median age (interquartile range [IQR]).

**Sampling site (site no.)**	**Origin of camels**	**Heartwater-like disease reported**	**Animals sampled (males/females)**	**Median age in years (IQR)**
**Isiolo (15)**		No	143 (0/98)†	9 (5)††
**Athi River (16)**	Moyale	Yes	115 (33/82)	10 (6.25)
	Bangale	No	51 (25/46)	10 (3.5)
	Pokot	No	5 (1/4)	11 (2)
	Marsabit	No	16 (11/5)	10 (4)
	Marigat	No	8 (4/4)	5 (1.75)
	Isiolo	No	35 (22/13)	6 (5)
	**Total**		230 (96/34)	10 (5)

†data available for 98/143 camels; ††data available for 93/143 camels
